# Comparative Transcriptome Analysis of Temperature-Induced Green Discoloration in Garlic

**DOI:** 10.1155/2018/6725728

**Published:** 2018-12-02

**Authors:** Ningyang Li, Zhichang Qiu, Xiaoming Lu, Bingchao Shi, Xiudong Sun, Xiaozhen Tang, Xuguang Qiao

**Affiliations:** ^1^Key Laboratory of Food Processing Technology and Quality Control in Shandong Province, College of Food Science and Engineering, Shandong Agricultural University, No. 61, Daizong Road, Tai'an, Shandong Province 271018, China; ^2^State Key Laboratory of Crop Biology, College of Horticulture Science and Engineering, Shandong Agricultural University, No. 61, Daizong Road, Tai'an, Shandong Province 271018, China

## Abstract

Green discoloration is one of the most important problems that cause low quality of product in the processing of garlic, which can be induced by low-temperature stress. But the mechanism of low temperature-induced green discoloration is poorly understood. In the present study, the control garlic and three low temperature-treated garlic samples (stored at 4°C with 10, 15, and 40 days, respectively) were used for genome-wide transcriptome profiling analysis. A total of 49280 garlic unigenes with an average length of 1337 bp were de novo assembled, 20231 of which were achieved for functional annotation. When being suffered from 10, 15, and 40 days of low-temperature treatment, an increased degree of discoloration was observed, and a total of 4757, 4401, and 2034 unigenes showed a differential expression, respectively. Finally, 5923 differentially expressed genes (DEGs) were found to respond to the low-temperature stress, of which 3921 were identified in at least two treatments. Among these stress-responsive unigenes, there were large numbers of enzyme-encoding genes, which significantly enriched the pathway “proteasome,” many genes of which are potentially involved in the garlic discoloration, such as 7 alliinase-encoding genes, 5 *γ*-glutamyltranspeptidase-encoding genes, and 1 *δ*-aminolevulinic acid dehydratase-encoding gene. These stress-responsive enzyme-encoding genes are possibly responsible for the low-temperature-induced garlic discoloration. The identification of large numbers of DEGs provides a basis for further elucidating the mechanism of low-temperature-induced green discoloration in garlic.

## 1. Introduction

Garlic (*Allium sativum*) is one of the most widely used food ingredients worldwide and also possesses excellent medicinal benefits. To provide conveniences for consumers, the bulbs of garlic are processed as products with various forms, such as powder, granules, puree, minced paste, and oleoresin. However, the garlic processing frequently causes a green discoloration, resulting in the loss of visual appeal and commercial value of garlic products [1]. Mechanistic studies indicated that green discoloration of garlic is a complex, multistep process beginning with the alliinase-catalyzed conversion of isoalliin and other S-substituted cysteine derivatives (alliin and methiin) to the corresponding sulfenic acids [2–4]. Subsequent reactions of the sulfenic acids result in the generation of 1-propenyl-containing thiosulfinates (including cepathiolanes), acting as a color developer to react with certain amino acids to produce color precursors of 3,4-dimethylpyrrole. The color precursors further react with allicin and various (thio) carbonyls to produce color compounds that are the hallmarks of garlic discoloration [4, 5]. In addition, recent studies have suggested that the membrane permeability of garlic cells also plays a key role in the greening process [6]. This is consistent with the finding that alliinase and isoalliin are physically separated from each other under normal circumstances but would only come into contact upon the injury of garlic tissues [7, 8]. Therefore, there are a large number of metabolites and enzymes, including isoalliin, alliinase, and unknown compounds, involved in the formation of green discoloration.

Garlic discoloration can be induced by some environmental stresses, such as low temperature, acetic acid compound, and various amino compounds [3, 9–11]. However, garlic has a giant genome (about 16 Gb), leading to the fact that little genetic and genomic information can be obtained in this crop. Therefore, the exact mechanism about these environmental factors inducing garlic greening is poorly understood.

In recent years, the next-generation sequencing (NGS) technology has emerged to offer a cost-effective and powerful tool for sequence determination [12, 13]. Transcriptome analysis by NGS is rapid, inexpensive, and unlimited by genomic complexity and has been widely used as a primary tool in many researching areas, including gene discovery, SSR marker development, and investigation of the domesticated patterns of crops, particularly in the characterization of the gene expression profiling [14–16]. The garlic transcriptome has been de novo assembled by several previous studies, and large numbers of expressed genes have been discovered [17, 18]. These works extended our knowledge in understanding of expression profiles, regulation, and networks of important traits of garlic, and thus they offered a new sight for garlic study.

In this study, to detect the potential mechanism of garlic discoloration caused by low-temperature stress, three low-temperature-treated garlic samples with different times which resulted in different degrees in discoloration and control garlic were exploited for genome-wide transcriptome profiling analysis. The characterization of expression profiling and identification of differentially expressed genes (DEGs) would provide a basis for further elucidating the formation mechanism of green discoloration in garlic.

## 2. Materials and Methods

### 2.1. Materials

Freshly harvested garlic cloves were used for the green discoloration and transcriptome analysis. Three treated groups (DS1, DS2, and DS3) and one control group (DS0) were set up. Each group contained four replications, and each replication included three cloves. Four replications of DS0, DS1, DS2, and DS3 were stored at 4°C for 0, 10, 15, and 40 days, respectively. After treatment, the cloves of three replications of each group were individually frozen in liquid nitrogen and then stored at −80°C until use for sequencing. The residual one replication of each group was used for the green discoloration.

To measure the extent of discoloration, 20 g of garlic cloves were used and the green sprouts were removed to minimize potential spectrometric interference. The sprout-free garlic samples were minced, mixed thoroughly with 5 mL of 2% citric acid solution, and incubated at 80°C for 30 min. The resultant mixture was cooled to room temperature, and then 95% ethanol was added to a final volume of 50 mL, followed by incubation at 4°C for 24 h. The garlic extract was then centrifuged at 12000 ×*g* for 5 min, and the absorbance of the supernatant was measured using an ultraviolet-visible spectrometer (Beckman, Michigan, USA) at 440 nm and 590 nm, respectively. The extent of green discoloration was represented by multiplying the absorbance at 440 or 590 nm by 10.

### 2.2. cDNA Library Construction and RNA Sequencing

Twelve samples from the four groups were used to extract total RNA using the RNeasy Plant Mini Kit (Qiagen, Germany) according to the manufacturer's instructions. The obtained RNA was quantified by NanoDrop 2000 (Thermo Fisher Scientific, USA) and Qubit 2.0 RNA Broad Range Assay Kit (Invitrogen, USA). The RNA integrity was assessed using an Agilent Bioanalyzer 2100 (Agilent Technologies, USA). An RNA integrity number (RIN) greater than or equal to 8 was considered to be useful. Subsequently, the library of each sample was constructed using the TruSeq RNA Sample Preparation Kit (Illumina, USA) and SuperScript II Reverse Transcriptase (Invitrogen, USA) according to the manufacturers' recommended protocols. Paired-end sequencing was then performed using the Illumina sequencing platform (HiSeq™ 4000) according to the manufacturer's instructions (Illumina, San Diego, CA). The quality of all sequences (in FASTQ format) was assessed by FastQC [19]. Adaptors and low-quality bases were trimmed using Trimmomatic [20]. Reads with a Phred quality score over 30 were used for the following transcriptome assembly.

### 2.3. Transcriptomic Analysis

The clean reads were further assembled using Trinity (version 2.4.0) [21] with min_kmer_cov set to 5 and other parameters to default values. All possible open reading frames within the assembled transcripts were extracted using TransDecoder (https://github.com/TransDecoder). Transcripts missing a likely CDS were discarded, and all predicted CDS sequences were translated into protein sequences and clustered by CD-HIT (version 4.6.6) [22] with 95% global sequence identity. Only the unique transcript with CDS sequence was designated as a unigene. The longest sequence in each cluster was transferred to the final data set. The translated protein sequences of all ORFs were annotated by performing BLAST searches against the NCBI nonredundant protein database (NR), Swiss-Prot, Gene Ontology (GO), Kyoto Encyclopedia of Unigenes and Genomes (KEGG), eggNOG, and protein family (PFAM) databases with e-value threshold set to 1*e* ^−5^.

### 2.4. Identification and Quantization of Gene Expression Levels

Transcript abundances were separately estimated by RSEM (version 1.3.0) [23] for each sample. Differential gene expression analysis was performed using DEGseq2, an R package [24]. A unigene was considered differentially expressed between two garlic groups if the criteria of the *Q* value < 0.05 and fold change > 2 were met. GO and KEGG enrichment analyses were performed using the topGO package [25] and KOBAS (version 3.0) [26], respectively.

### 2.5. Quantitative Reverse-Transcription Polymerase Chain Reaction (qRT-PCR) Validation

Twenty candidates were chosen from the top 100 most differentially expressed unigenes for qRT-PCR analysis. PCR primers were designed using Primer Premier 5.0 software and summarized in Table S1. Total RNA was reverse-transcribed into single-stranded cDNA using Takara PrimeScript RT Reagent Kit with gDNA Eraser (Clontech laboratories, USA). qRT-PCR was set up using SYBR Premix Ex Taq polymerase (Clontech laboratories, USA) following the manufacturer's recommended protocol and conducted on an Applied Biosystems 7500 Fast Real-Time PCR System (Thermo Fisher Scientific, USA). All PCR reactions were performed in triplicate, and the relative expression of each unigene was calculated using the 2^–ΔΔCT^ method with actin as the reference [27].

## 3. Results

### 3.1. Investigation of Green Discoloration in Four Groups

**Figure 1:**
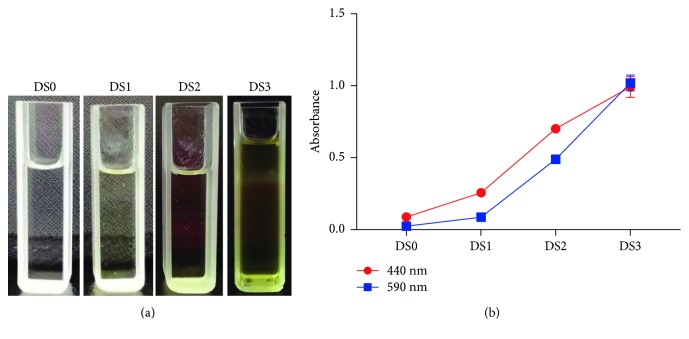
Assessment of greening in different experiment groups. (a) Appearance of pigment extracts prepared from DS0 to DS3. (b) Colorimetric measurement of pigment content at 440 nm and 590 nm.

As shown in Figure 1(a), with the extension of the treatment time, the samples DS1, DS2, and DS3 displayed an increase in the extent of greening, whereas the control DS0 served as the negative control in which discoloration had not been induced. The progression of greening from DS0 to DS3 was also confirmed by the detection of an increasing amount of green pigments as evidenced by colorimetry (Figure 1(b)).

### 3.2. Transcriptome Sequencing, Assembly, and Annotation

Three repeats of DS0, DS1, DS2, and DS4 were individually performed for Illumina sequencing. After quality filtering, we obtained an average of 70.48, 74.97, 72.05, and 69.74 million clean reads from DS0, DS1, DS2, and DS3 samples, respectively, representing 129 Gb of sequencing data in total (Table S2). De novo assembly of the reads by Trinity generated 49280 unigenes with an average length of 1337 bp. Among these 49280 unigenes, 14374 (29.17%), 8647 (17.55%), 4405 (8.94%), 20210 (41.01%), 3527 (7.16%), and 9127 (18.52%) showed significant similarity to known proteins in the eggNOG, GO, KEGG, NR, PFAM, and SwissProt databases, respectively. In total, there were 20231 (41.05%) unigenes that were achieved for functional annotation in at least one database (Table S3).

### 3.3. Identification of DEGs

**Figure 2:**
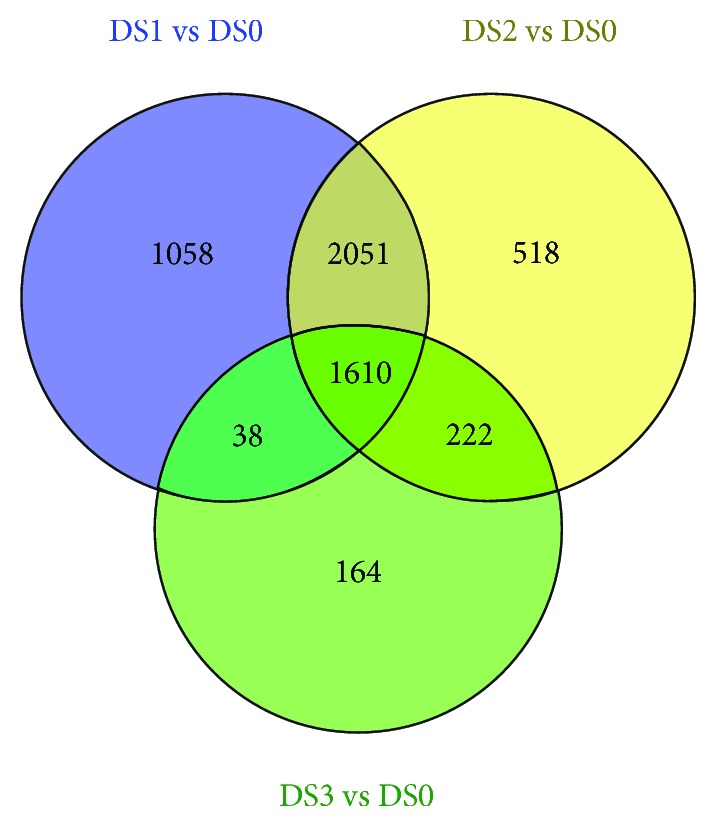
Venn diagram of the DEGs identified in three treatments.

**Figure 3:**
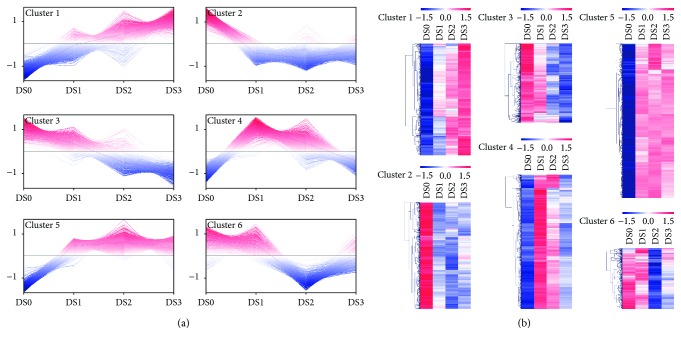
Clustering of DEGs based on their expression pattern control and three treatments. (a) The expression trend of six clusters. (b) The hot map of gene expression level in each cluster.

When garlic was stored at 4°C for 10 (DS1), 15 (DS2), and 40 days (DS3), there were 4757, 4401, and 2034 unigenes that showed differential expression, respectively (Figure 2, Table S4). Among these DEGs, 3921 unigenes were identified in at least two treatments, including 2051 in D1 and D2, 38 in D1 and D3, 222 in D2 and D3, and 1610 in all three treatments, respectively (Figure 2, Table S4). In total, we identified 5923 DEGs, including 7 allinase-encoding genes, 5 *γ*-glutamyltranspeptidase-encoding genes, and 1 *δ*-aminolevulinic acid dehydratase gene (Table S4). We also analyzed the expression trends of these 5923 DEGs, and the result showed that they could be classified into six distinct clusters (Table S4), and each group showed a similar expression pattern (Figure 3). DEGs in three clusters (clusters 1, 4, and 5) exhibited a trend of upregulated expression, whereas the expression trends of DEGs in the other clusters (clusters 2, 3, and 6) were downregulated. Therefore, under our experimental conditions, although the expressed difference of the 4313 out of 5923 DEGs was only significant in one or two of three treatments, the trends of continuously up- or down regulated expression in all three treatments could be observed (Figure 3).

### 3.4. Validation of the Differential Expression by qRT-PCR

**Figure 4:**
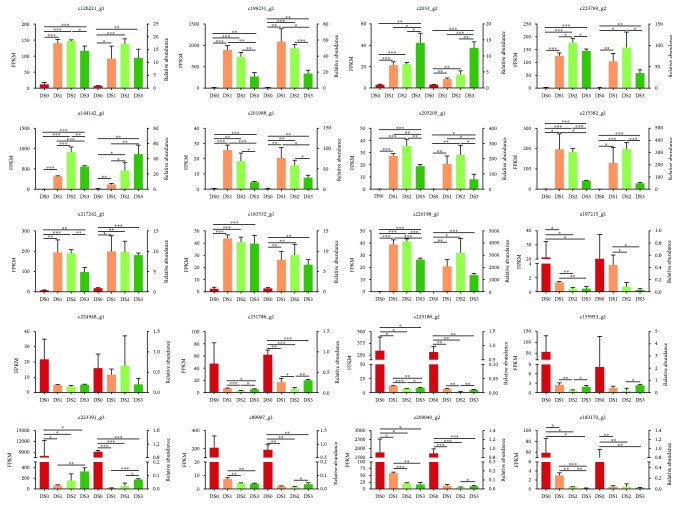
qRT-PCR validation of DEGs. All experiments were performed in triplicate. ^∗^ *P* < 0.05; ^∗∗^ *P* < 0.01; ^∗∗∗^ *P* < 0.001.

To validate the expression profiling by Illumina sequencing, the expression levels of twenty DEGs (11 upregulated and 9 downregulated) involved with beta-glucosidase, pectate lyase, aquaporin, vacuolar processing, heat shock protein, chaperone, and glutamate-cysteine ligase were selected for qRT-PCR analysis (Table S1). The result indicated that the trends of the expression change of all unigenes analyzed by qRT-PCR were identical to those by Illumina sequencing, except for c224948_g1 (Figure 4). However, the change folds of the expression levels of these DEGs checked by qRT-PCR have slight difference with those by Illumina sequencing. These results confirmed that our RNA sequencing and quantitation results were accurate and could be used for subsequent functional analysis to identify candidates mechanistically implicated in garlic greening.

### 3.5. Enrichment Analysis of DEGs

**Figure 5:**
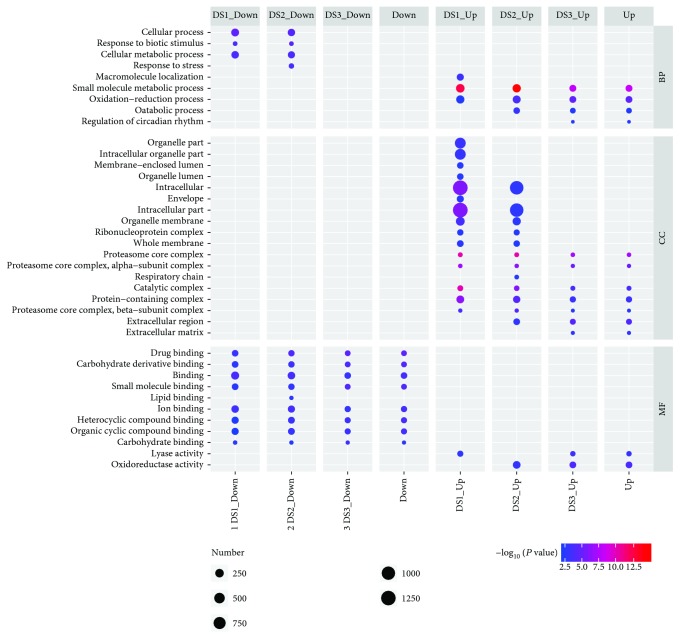
GO enrichment analysis of up- and downregulated DEGs in garlic greening samples.

To determine the function categories that DEGs were involved in, the GO categories enriched by DEGs were analyzed. The functional enrichment analysis indicated that upregulated DEGs and downregulated DEGs in garlic greening samples were significantly enriched in different GO terms (Table S5). It was found that coupregulated DEGs are highly related to cellular component functions and metabolic and catabolic functions, such as organelle part/lumen, intracellular, membrane, organelle envelope, protein-containing complex, proteasome core complex, small molecule metabolic process, and oxidation-reduction process. Other functions such as lyase activity, oxidoreductase activity and catalytic activity were also enriched. Surprisingly, when the codownregulated DEGs were analyzed, the functions of binding, cellular metabolic process, and response-related functions were significantly enriched, such as carbohydrate derivative binding, small molecule binding, heterocyclic/organic compound binding, response to stress, and biotic stimulus (Figure 5). The GO enrichment analysis indicated that the genes coupregulated in greening process are involved in divergent biology processes and cellular component functions, especially in the structural maintenance of organelle and intracellular but not restricted to stress-related.

The biological pathways influenced by DEGs were determined by KEGG enrichment analysis. Our result showed that one pathway, proteasome (ID: ko03050) was dramatically enriched by DEGs of DS1 (*Q* value = 0.015), and another pathway, glycolysis/gluconeogenesis (ID: ko00010) was markedly enriched by DEGs of DS3 (*Q* value = 0.014). None of pathways showed significant enrichment by DEGs of DS2.

## 4. Discussion

Comparative transcriptomics analysis has demonstrated enormous utilities in helping researchers understand complex biological processes in plants. Villacorta-Martin et al. explored the transcriptomic changes that occurred in the meristems of lily bulbs at several time points during cold exposure and identified 9872 dysregulated unigenes from 42430 unigenes during the first two weeks of cold exposure [28]. Similarly, Chaturvedi et al. found 3307 upregulated and 4996 downregulated unigenes in the internal bud (IB) of garlic cloves at 33°C compared to 2°C treatment. They also found 5703 upregulated and 8444 downregulated unigenes in storage leaf (SL) at 33°C compared to 2°C treatment [29]. Our current study aimed at generating mechanistic insight into garlic greening via systematic transcriptomic profiling. We successfully identified 49280 unigenes from a total of 87570 unique transcripts, from which 5923 DEGs were found to be differentially expressed in garlic samples undergoing discoloration. Among them, the most upregulated unigenes in the greening garlic cloves were compared to the control including *β*-glucosidases, pectatelyases, NRT1/PTR family proteins, aquaporins, and various proteases, whereas the most downregulated candidates were shown to consist primarily of chaperones, heat shock proteins, pectins, and metabolic enzymes. Subsequent functional analysis suggested that the transcriptomic alterations observed in the garlic specimens undergoing discoloration could signify changes in various metabolic, stress response, and cellular component functions.

Some of the differentially expressed unigenes in our transcriptomic study unearthed have been validated in previous studies. For example, our results indicated that *γ*-glutamyltranspeptidase (GGT, c187783_g1, c199606_g1, c222769_g1, and c222769_g2) was among one of the most upregulated unigenes in greening garlic cloves. Previously, Li et al. reported that GGT activity was significantly augmented in garlic bulbs stored at 4°C, which could be reversed by incubation at 35°C [30]. The striking similarity between the trend of GGT activity and that of total thiosulfinate level prompted further investigation into the putative role of the enzyme in the biosynthesis of color developers responsible for garlic greening. Indeed, GGT was subsequently shown by Yoshimoto and coworkers to catalyze the deglutamylation of *γ*-glutamyl-S-(1-propenyl)-L-cysteine, a hypothetical isoalliin precursor [31]. Therefore, the upregulation of GGT could be an early indicator of activated isoalliin production and garlic greening.

It has been proposed that certain stress conditions such as low temperature could cause injury to vacuolar membranes in plant cells [32, 33]. This allows alliinase to be released from the vacuoles and come into contact with its substrate isoalliin present in the cytosol [7, 8]. Consistent with these findings, Bai et al. reported that treatment of garlic cloves with 5% (*v*/*v*) acetic acid resulted in significant tonoplast damage and a concomitant increase in the concentration of thiosulfinates [6]. The differential proteomic signatures between the greening groups and the control group in our current study also provided several clues to the possible involvement of vacuole disruption. For example, c199231_g1 and c221124_g1, annotated as a possible aquaporin TIP2-2 homolog, was shown to be consistently among the top 10 most upregulated unigenes. Aquaporins form the water channels on cellular and vacuolar membranes and are mechanistically implicated in the regulation of osmotic stress signals [34]. Not surprisingly, expression of aquaporins is often enhanced by low temperature as the change in water density and solubility can result in altered osmotic pressure [35]. Increased expression of an aquaporin SlTIP2-2 was found to enhance the osmotic water permeability of *Arabidopsis* mesophyll protoplasts, suggesting underlying structural changes in the vacuolar membranes [36]. Additional evidence implied that possible vacuolar damage involved in the increased expression of various proteases, particularly several vacuolar-processing enzyme (VPE) homologs (c217242_g1, c176154_g2, c176154_g3, and c200027_g3). Upregulation of VPEs has been shown to play a crucial role in the stress response of *Arabidopsis* plants by promoting the destruction of vacuolar structures, leading to the release of diverse hydrolytic enzymes into the cytosol [36–38]. Therefore, it seemed plausible that cold stress-induced osmotic change and activation of processing proteases could be a key contributing factor to the leakage of alliinase from vacuole to cytosol. Of course, further examination would be needed to determine the exact localization patterns and functions of the upregulated proteolytic enzymes that we identified.

Many of the differentially expressed unigenes uncovered in our current study could have been the result of low-temperature storage. NRT1/PTR family proteins (c220198_g1, c225222_g1, c208304_g1, c169637_g1, c212989_g1, c228990_g1, c204357_g1, c210835_g1, c229656_g3, c75070_g1, c207568_g1, and c214187_g1) are involved in nitrogen transport [39] and have been reported to undergo upregulation in response to cold stress in *Saussurea involucrata* [40]. Cold stress has also been known to modulate sugar metabolism and alter the composition of plant cell wall [41]. This was consistent with our identification of several putative *β*-glucosidases (c144142_g1, c223769_g2, c183532_g1, c201088_g1, c155953_g1, c197248_g1, c230818_g2, c231722_g1, c231722_g3, c218711_g1, c155121_g2, c186294_g1, c320855_g1, c176603_g1, c228932_g1, c218383_g2, c195036_g1 and c178982_g1) and pectatelyases (c205205_g1, c215382_g1, c215382_g2, c193411_g2, c211032_g1, c207990_g2, and c207990_g1) that showed aberrant expression levels in comparison with the control. Altered levels of *β*-glucosidase and pectatelyase induced by cold storage or other types of abiotic stress have also been reported elsewhere [42–44]. On the other hand, several of the most downregulated unigenes in the greening groups were shown to be members of the lectin superfamily. Lectins are a large group of internally heterologous carbohydrate-binding proteins with functions that have yet to be fully characterized [45]. Nevertheless, it has been suggested that lectins might play an important role in stress regulation in higher plants [46]. Furthermore, our study also revealed that a variety of chaperones and heat shock proteins were downregulated, which was very similar to the result of plant response to cold stress. Although it is well known that low-temperature storage was an effective method to induce garlic greening, further studies would be required to unambiguously determine whether the differentially expressed unigenes that we identified were causally involved in the greening process or were merely dysregulated due to cold acclimatization of garlic.

## Data Availability

The RNA-Seq data used to support the findings of this study have been deposited in the Short Read Archive at the National Center for Biotechnology Information (study accession SRP148664).

## Supplementary Materials

Supplementary Materials Supplementary data to this article can be found online and in Data Availability. 332 Supplementary Table S1: qRT-PCR primers of selected unigenes. 333 Supplementary Table S2: summary of transcriptome sequencing. 334 Supplementary Table S3: the function of unigenes annotated. 335 Supplementary Table S4: DEGs identified in three treatments. 336 Supplementary Table S5: GO terms enriched by DEGs.
