# Supplementary material for: Comparative Transcriptome Analysis of Temperature-Induced Green Discoloration in Garlic
Source: Int J Genomics. 2018 Dec 2;2018:6725728. doi: 10.1155/2018/6725728 (PMC6304921; doi:10.1155/2018/6725728)
Supplement: Supplementary Materials — Supplementary data to this article can be found online and in Data Availability. 332 Supplementary Table S1: qRT-PCR primers of selected unigenes. 333 Supplementary Table S2: summary of transcriptome sequencing. 334 Supplementary Table S3: the function of unigenes annotated. 335 Supplementary Table S4: DEGs identified in three treatments. 336 Supplementary Table S5: GO terms enriched by DEGs. [file 6725728.f1.zip › Supplementary Material.docx]

**Supplementary Material**

**Journal: *International Journal of Genomics.***

**Title:** **Comparative transcriptome analysis of temperature-induced green discoloration in garlic**

**Authors: Ningyang Li, Zhichang Qiu, Bingchao Shi, Xiaoming Lu, Xiudong Sun,Xuguang Qiao, Xiaozhen Tang**

**The following is included as Supporting Information for this paper:**

**Supplementary Tables**

Table S1: qRT-PCR Primers of selected unigenes.

Table S2: Summary of transcriptome sequencing.

Table S3: The function of unigenes annotated.

Table S4: DEGs identified in three treatments.

Table S5: GO terms enriched by DEGs (P< 0.001)
